# Case Report: Fever of unknown origin with hemophagocytic lymphohistiocytosis and intestinal hemorrhage—a successfully treated case of severe visceral leishmaniasis

**DOI:** 10.3389/fmed.2025.1663551

**Published:** 2025-10-02

**Authors:** Hongmei Wang, Jiajun Li, Wenxiang Huang

**Affiliations:** ^1^Department of Geriatrics, The First Affiliated Hospital of Chongqing Medical University, Chongqing, China; ^2^Department of Infectious Diseases, The First Affiliated Hospital of Chongqing Medical University, Chongqing, China

**Keywords:** visceral leishmaniasis, intestinal hemorrhage, hemophagocytic lymphohistiocytosis, metagenomic next-generation sequencing, amphotericin B deoxycholate

## Abstract

In recent years, some cases of severe visceral leishmaniasis (VL) in immunocompetent adults have gradually been reported. Hemophagocytic lymphohistiocytosis (HLH) and intestinal hemorrhage are two rare complications in patients with VL. Without treatment, the mortality rate of such patients is extremely high. We report a case of a 31-year-old immunocompetent male who initially presented with fever of unknown origin (FUO), later developed HLH and experienced multiple episodes of life-threatening intestinal hemorrhage. The diagnosis of visceral leishmaniasis was confirmed through metagenomic next-generation sequencing (mNGS). The patient was successfully treated with amphotericin B deoxycholate (AmB-D) and supportive care. During the two-year follow-up period, no new complications were found. This case highlights the value of mNGS in the diagnosis of complex infectious diseases and emphasizes the clinical significance of the multidisciplinary collaborative model for patients with VL and complex complications. It can provide a reference for the early diagnosis and comprehensive treatment of severe VL.

## 1 Introduction

Visceral leishmaniasis (VL), also known as kala-azar, is the second most fatal parasitic disease in the world after malaria. VL is caused mainly by *Leishmania infantum (L. infantum)* and *Leishmania donovani (L. donovani)*, which are transmitted among mammalian hosts through sanflies (1). VL remains endemic in more than 60 countries: Brazil, Ethiopia, India, Kenya, Somalia, South Sudan, and Sudan have reported more than 90% of the cases worldwide (2). The reported cases in mainland China were mainly in six provinces: Shanxi, Shaanxi, Gansu, Sichuan, Xinjiang and Inner Mongolia (3).

The clinical manifestations of VL are diverse and nonspecific. Persistent irregular fever, splenomegaly, and anemia typically constitute the initial symptoms. As the disease progresses, symptoms may include progressive splenomegaly, pancytopenia, hyperimmunoglobulinemia, weight loss, coinfections, and multiorgan failure, etc. (1, 2). VL can affect any organ of the human body, including the gastrointestinal tract, peritoneum, lungs, and pleural space (4). However, cases of intestinal bleeding caused by VL in immunocompetent adults are extremely rare. VL is indistinguishable from many other diseases, including hematologic malignancies, hemophagocytic lymphohistiocytosis (HLH), and connective tissue diseases, therefore it is easily misdiagnosed or underdiagnosed in clinical practice. Without timely and appropriate treatment, VL can progress to secondary HLH, a rare and life-threatening complication for which Epstein–Barr virus (EBV) infection is thought to be the most common predisposing factor and is characterized by excessive immune activation and hyperinflammation. VL and HLH share overlapping clinical features, such as fever, hepatosplenomegaly, and cytopenia, which can lead to misdiagnosis (5–7). Microscopic examination of bone marrow, lymph node and spleen aspirates remains the most reliable method for confirming the diagnosis of VL. Currently, various PCR-based assays show promising applications with high sensitivity and specificity and allow for species identification (5).

Herein we report the case of a 31-year-old patient with no comorbidities or immunocompromising conditions who was admitted with FUO. Initial diagnostic efforts were inconclusive, and the patient’s condition progressed to HLH, accompanied by recurrent, life-threatening intestinal hemorrhage. Eventually, the mNGS identified Leishmania infection, confirming a diagnosis of VL. The patient underwent antiparasitic therapy with AmB-D and received multiple endoscopic hemostatic interventions. He recovered and was discharged in stable condition. Over a follow-up period exceeding 2 years, no recurrence was observed.

## 2 Case description

**Figure 1:**
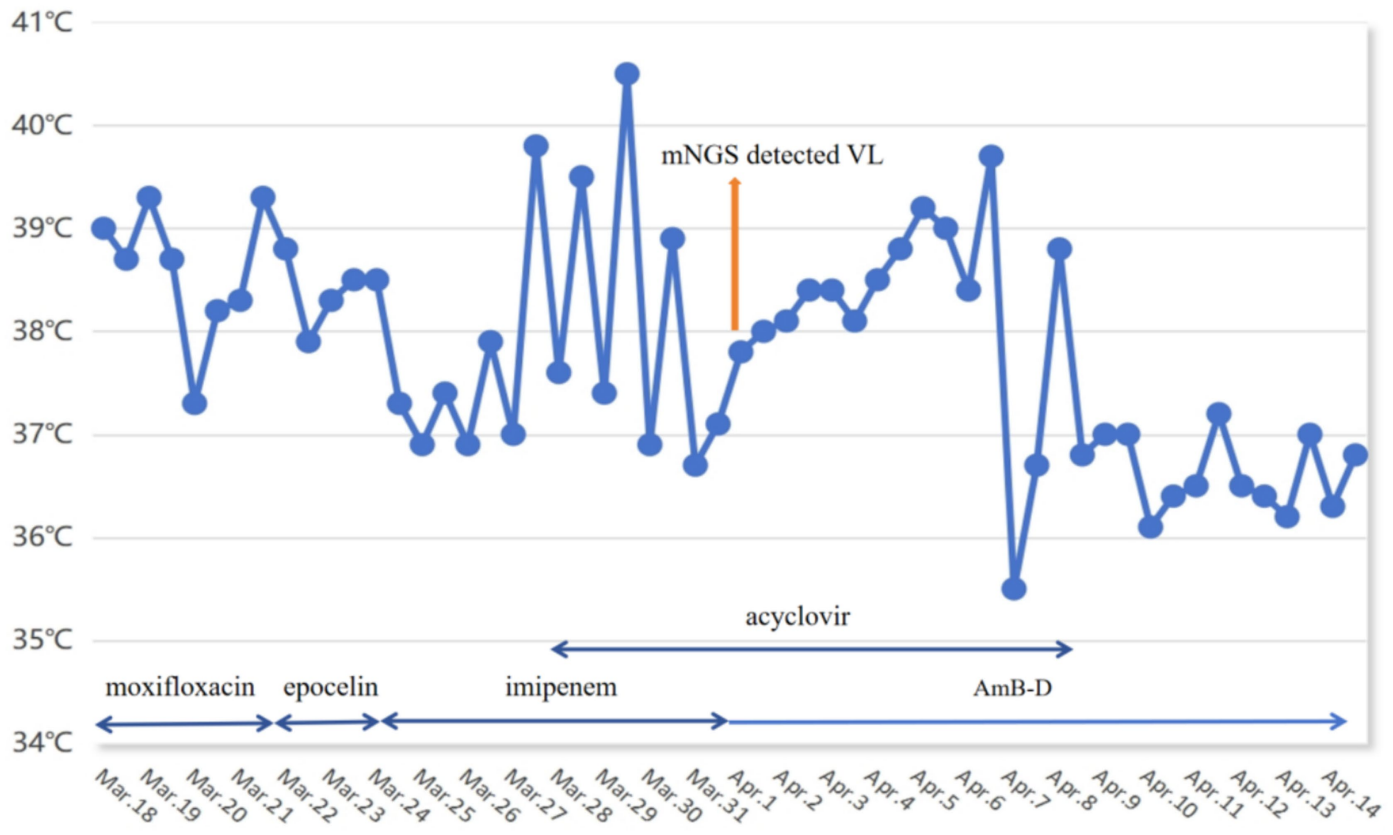
Changes of patient’s temperature and antibiotic treatment therapy.

A 31-year-old man was referred to our hospital on 18 March 2022 due to recurrent fever accompanied by dry cough for 22 days. The maximum temperature of 40.5 °C occurred at night and the type is continued fever (Figure 1). It was accompanied by dry cough, runny nose, fatigues, nausea and vomiting. The patient received 10 days of antibacterial and antiviral therapy at a local hospital, but his symptoms did not improve. He reported a decrease in body weight of approximately 10 kg in the previous 2 months. Additionally, 6 months ago, he had traveled to Xinjiang, which is one of the endemic areas of VL in China. He did not have any underlying disease and denied any exposure to radioactive or toxic substances. Physical examination revealed no significant abnormalities.

The patient was admitted with a diagnosis of FUO. As part of the differential diagnosis, he underwent a series of laboratory tests after admission (Table 1), with the following results: leukocyte count 2.9 × 10^9^ cells/L, neutrophil percentage 80.5% and neutrophil count 1.6 × 10^9^ cells/L; lymphocyte percentage 15.6%, hemoglobin content 85 g/L, platelet count 97 × 10^9^ cells/L, fibrinogen 1.44 g/L, serum ferritin (Ft) 76,865 ng/mL, serum IL-2 receptor 7,500 U/mL, alanine aminotransferase (ALT) 480 U/L and aspartate aminotransferase (AST) 601 U/L; natural killer cell 41 cells/μL, EBV-DNA 9.79 × 10^3^ IU/mL. The tuberculosis T-cell spot test (T-SPOT. TB) was positive. Tests for antibodies specific for HIV, syphilis, toxoplasma gondii, herpes virus, and rubella virus were all negative. In addition, his sputum, urine, faecal, blood and bone marrow cultures were normal. Meanwhile, he perfected whole-body FDG positron emission tomography/ computed tomography (FDG PET/CT), suggesting an splenomegaly and multiple sites of lymph node hyperplasia.

**Table 1:** Data of laboratory examinations.

Items	Reference range	Mar.18	Mar.21	Mar.24	Mar.27	Mar.30	Apr.2	Apr.5	Apr.8	Apr.13	Apr.18	Apr.24	May. 10	Mat. 26
WBC (10^9^/L)	3.5–9.5	2.9	2.12	0.96	9.31	4.57	3.13	1.83	9.89	4.55	11.28	13.74	7.03	5.66
NEUT# (10^9^/L)	1.8–6.3	1.6	1.63	/	6.14	2.7	1.58	/	8.97	3.89	9.09	11.27	5.2	3.35
LYM# (10^9^/L)	1.1–3.2	0.43	0.34	/	1.12	0.37	1.41	/	0.57	0.49	1.16	0.55	1.25	0.67
RBC (T/L)	4.3–5.8	3.12	3.26	2.55	2.91	2.89	2.9	2.71	2.55	2.82	2.72	2.66	2.97	3.74
HGB (g/L)	130–175	89	94	72	83	82	86	80	74	83	82	85	109	123
PLT (10^9^/L)	85–300	97	121	198	80	50	135	113	70	45	212	214	212	210
IL-2R (U/mL)	223–710	>7,500				>7,500			>7,500	6,066	4,321	1987	1,005	451
FER (ng/mL)	30–400	76,865	80,239			17,325	22,193	36,659	36,200	9,262	2,495	1,569	533	520
D-Dimer (mg/L)	0–0.55	39.78	41.87		5.89	7.43	10.6	27.23	9.98	5.37	1.32	1.11		0.63
FBG (g/L)	1.8–3.8	3.24	2.31		1.52	1.44	1.76	1.85	1.52	1.22	1.53	1.57		1.76
K (mmol/L)	3.5–5.1	3.6	3.7	4.8	4.1	4.3	4.0	4.2	3.0	2.7	3.1	3.5	3.6	4.3
Na (mmol/L)	137–147	124	126	122	132	132	124	127	129	134	141	144	144	146
ALT (U/L)	9–50	400	307		112	116	67	167		46	134	42	22	18
AST (U/L)	15–40	601	369		130	226	109	202		22	258	16	14	12
ESR (mm/1 h)	0–20	35					8							
CRP (mg/L)	< 10	144		41.5			14.8				5.93			
ST-OB	−	+	+		−		+			−				−
EBV^a^-DNA (IU/mL)	<1*10^3^				9.79*10^3^		−						−	

WBC, white blood cell; NEUT, neutrophil; LYM, lymphocyte; RBC, Red Blood Cell; HGB, hemoglobin; PLT, platelet; IL-2R,serum IL-2 receptor; FER, ferritin; FBG, Fibrinogen; ALT, alanine aminotransferase; AST, aspartate transaminase; ESR, erythrocyte sedimentation rate; CRP, C-reactive protein; ST-OB, Stool Occult Blood; EBV, Epstein–Barr Virus; EBNA-IgG, Epstein–Barr Nuclear Antigen Immunoglobulin G; VCA-IgM, EBV Viral Capsid Antigen Immunoglobulin M; EA-IgA, EBV Early Antigen Immunoglobulin A; +, positive; −, negative; /, Undetectable.

a EBNA-IgG(+), VCA-IgM(−) and EA-IgA(+) in Mar.18.

**Figure 2:**
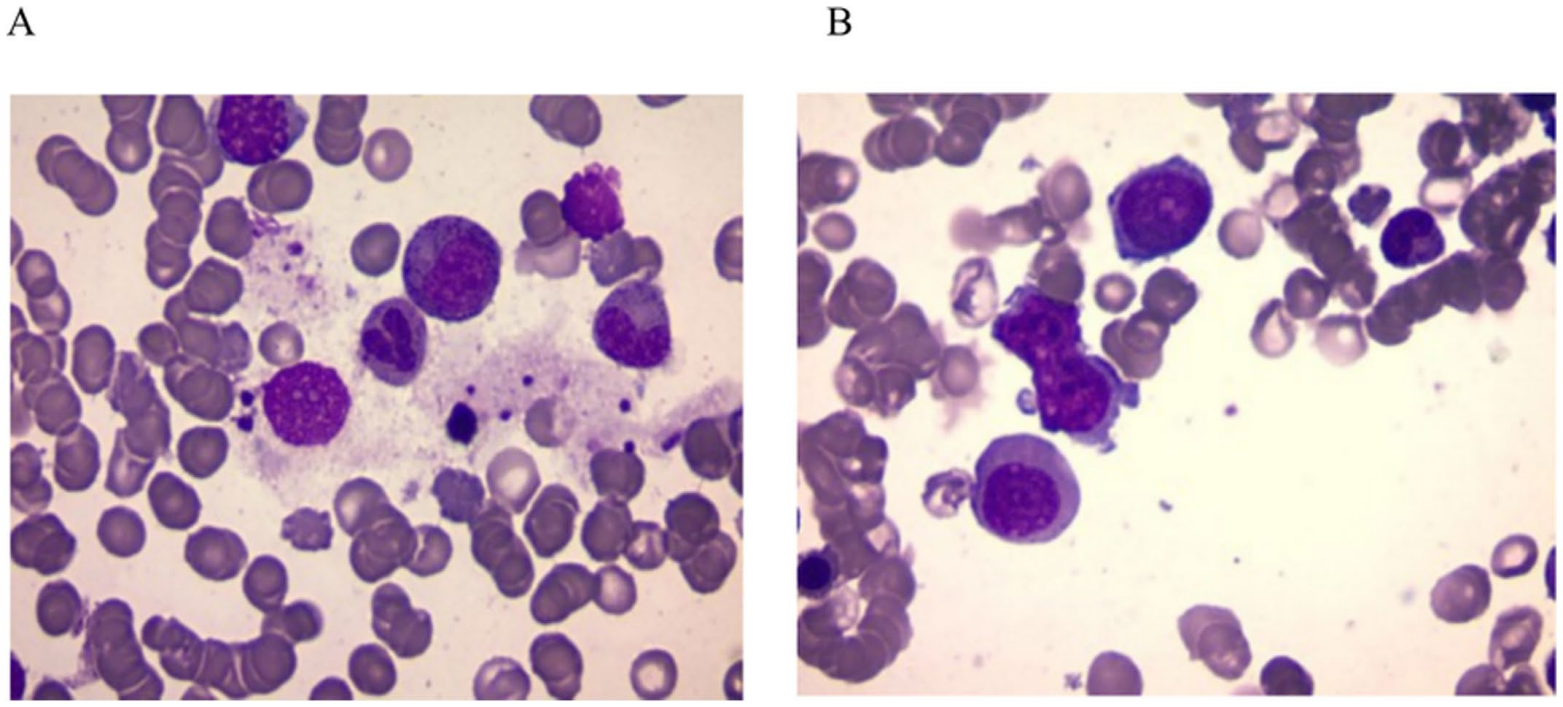
Bone marrow smear. **(A)** Phagocytosis of nucleated red blood cell and granulocyte by bone marrow macrophages. **(B)** Abnormal morphological blood cells. No amastigotes invading bone marrow macrophages were observed in both images.

**Figure 3:**
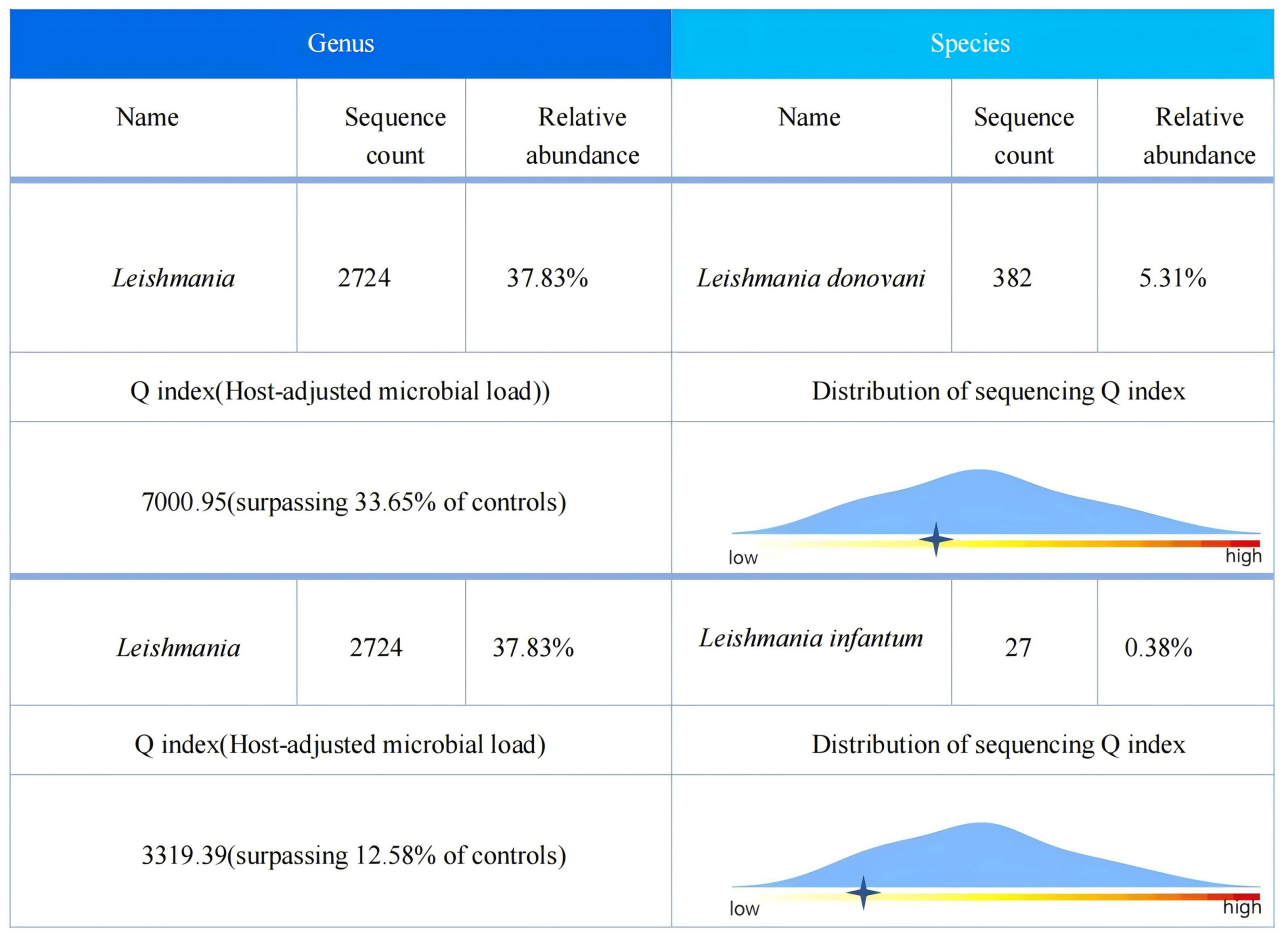
mNGS report of peripheral blood.

**Figure 4:**
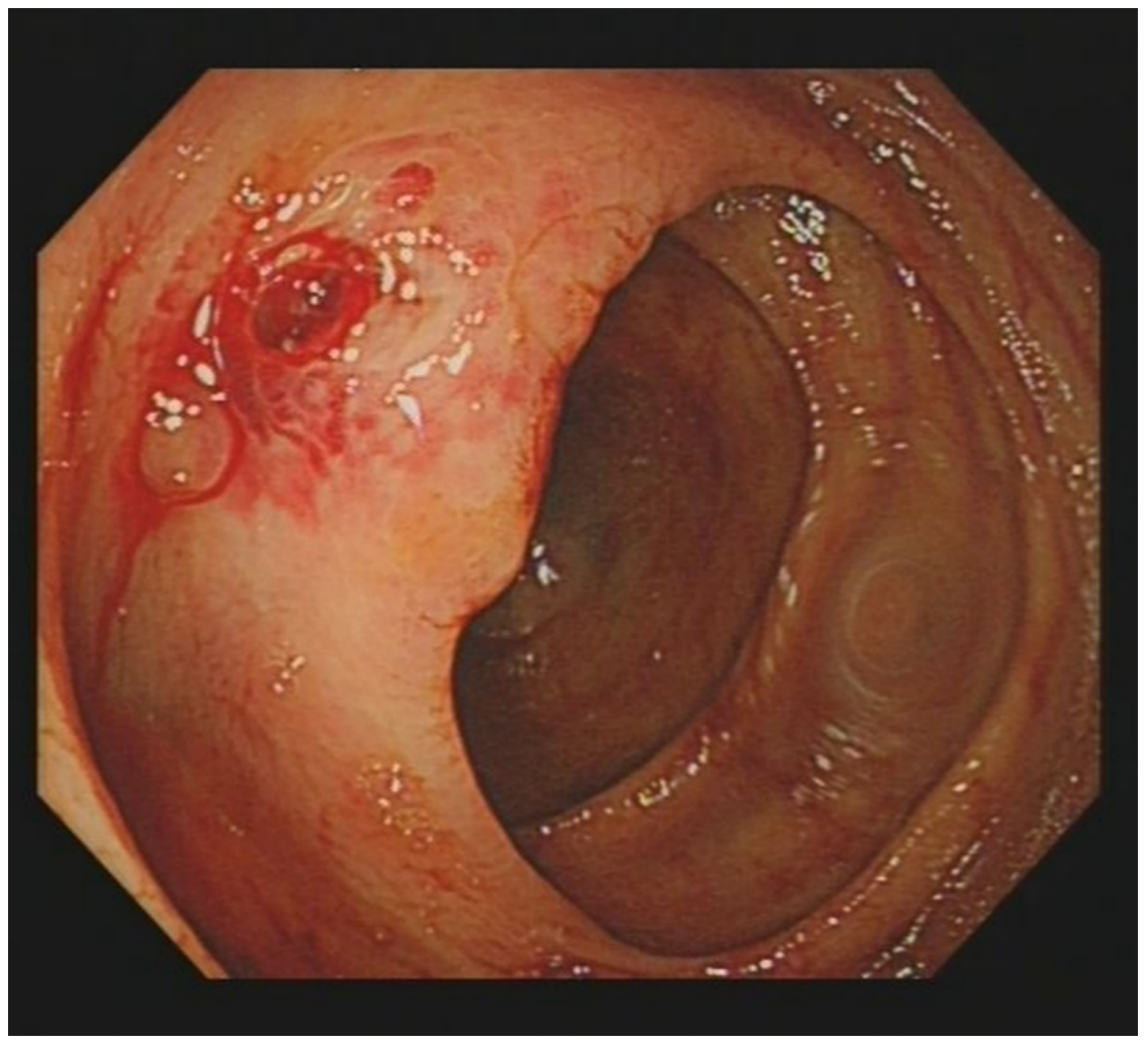
Colonoscopy revealed a ulcer with active bleeding on the ileocecal valve surface.

**Figure 5:**
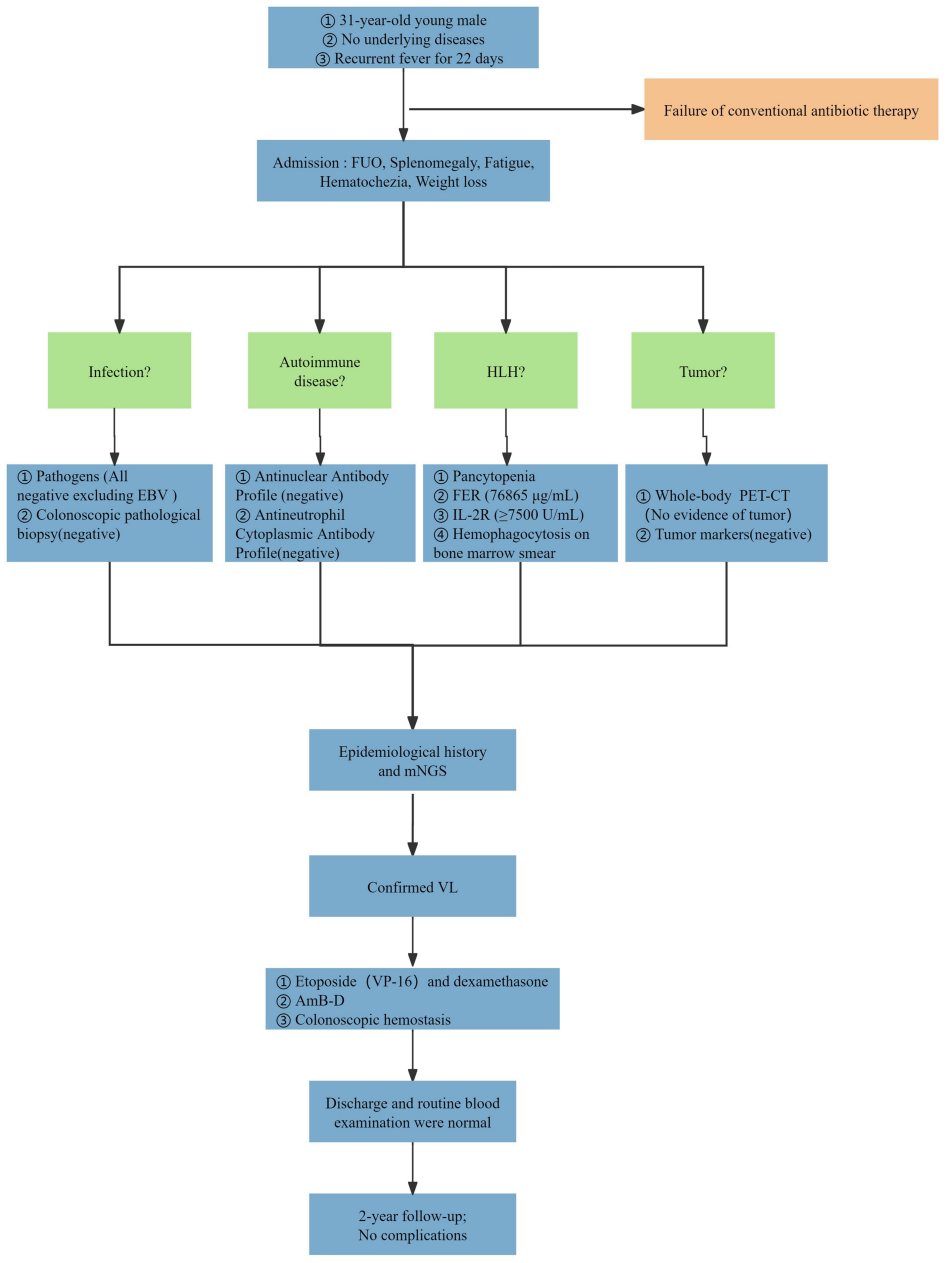
A diagnostic-therapeutic flowchart for the patient.

Given the presence of above results, a hematologic malignancy was suspected after consultation with hematologists. The patient subsequently underwent comprehensive bone marrow examination, including aspiration smears, biopsies, and flow cytometry analysis. A bone marrow smear revealed hypercellularity with evidence of active myeloproliferation, accompanied by the presence of hemophagocytosis, while no Leishmania amastigotes or other parasites or microorganisms were identified (Figure 2). Immunophenotyping of the bone marrow was conducted but did not reveal any immunophenotypic abnormalities. Finally, the mNGS of the peripheral blood samples showed the Leishmania spp. (2,724 reads) with a relative abundance of 37.83%, including *L. infantum* (27 reads) and *L. donovani* (382 reads) (Figure 3). Therefore the clinical diagnosis of kala-azar was made. Treatment with AmB-D was initiated on the day of diagnosis. Starting with an initial dose of 5 mg·d-1, the dosage was increased by 5 mg daily. At a dosage of 45 mg·d^−1^, the patient exhibited leukopenia, granulocyte deficiency, and recurrent hypokalemia; however his body temperature was normal, and these symptoms were considered related to adverse reactions of AmB-D. Consequently, the dosage of AmB-D was gradually reduced, ultimately maintaining the patient at 25 mg·d^−1^. On the 38th day, the mNGS still revealed Leishmania spp., but the reads decreased significantly. Thus another 15 days of AmB-D was used to intensify the efficacy with a total dosage of 1,385 mg in 53 days. In addition, the patient was diagnosed with Visceral Leishmaniasis-Associated Hemophagocytic Lymphohistiocytosis (VL-associated HLH). Therefore, dexamethasone and etoposide (VP-16) were combined for the treatment of HLH. In particular, the patient experienced recurrent episodes of bloody stools during his hospitalization. Colonoscopy revealed a surface ulcer with active bleeding in the ileocecal valve on three occasions (Figure 4). His symptoms significantly improved after undergoing endoscopic electrocoagulation hemostasis in collaboration with gastroenterologists and gastrointestinal surgeons. It is noteworthy that the patient also underwent colonoscopy at a local hospital prior to admission to our hospital, which revealed a lesion in the ileocecal region and a simultaneous biopsy, which showed chronic inflammation of the intestinal mucosa, but there was no black stool as well as blood at that time. Following aggressive treatment, there was a marked improvement in his clinical symptoms and laboratory indicators. He was discharged 68 days after admission. We conducted a two-year follow-up on the patient, and he is now in good overall condition with all biochemical parameters back to normal. A diagnostic-therapeutic flowchart is presented in Figure 5.

## 3 Discussion

VL is a severe parasitic disease primarily caused by *L. donovani* and *L. infantum*. The incubation period of VL usually ranges from weeks to years, and its clinical symptoms are diverse and nonspecific (6). Moreover, the features of many cases overlap with those of HLH or hematologic malignancies, making diagnosis difficult. Therefore, a history of living in or traveling to endemic areas is important. The patient has no history of long-term residence in areas endemic to VL. Notably, no evidence of Leishman-Donovan bodies was found in the bone marrow smear. Upon repeated questioning, the patient recalled a history of work in Xinjiang 6 months earlier, which prompted us to strongly recommend that the patient underwent peripheral blood mNGS to find the answer. Thus, it is critical to evaluate epidemiologic data several times over. Additionally, in circumstances where conventional diagnostic methods prove challenging, mNGS is recommended to identify potential infectious pathogens, thereby increasing diagnostic accuracy and reducing the risk of misdiagnosis and inappropriate treatment. In our case, both *L. infantum* and *L. donovani* were detected in the peripheral blood mNGS test. Due to the high homology between the gene sequences of these two pathogens, when sequences detected by mNGS simultaneously match both genus-specific sequences and species-nonspecific sequences, further sequence alignment may result in the reporting of two distinct Leishmania parasites (7). In our case, we should prioritize the species with higher sequence reads: *L. donovani*. The second mNGS detected only *L. donovani*, which may support our hypothesis. However, the molecular biological differences between these two parasites do not affect subsequent treatments. On the basis of the VL diagnosis, the patient’s condition significantly improved following treatment with AmB-D.

VL is a systemic disease that not only affects the mononuclear phagocyte system, but may also affect almost any organ in the human body. Intestinal involvement is generally extremely rare; when it occurs, the duodenum and esophagus are most commonly affected (8). In our case, the patient experienced recurrent intestinal hemorrhage during his hospitalization. First, we needed to rule out the possibility that the bleeding could be due to intestinal tuberculosis, tumors, or autoimmune diseases. Therefore, we reanalyzed the histopathology slides, which revealed chronic inflammation, negative TB-DNA and no evidence of tumor or autoimmune disease. Although the pathologic tissue biopsy did not reveal direct evidence of intestinal mucosal lesions, we need to consider that the intestinal mucosal lesions were due to the invasion of Leishmania protozoa, and the intestinal bleeding may have been induced by the biopsy procedure. Previous studies have shown that VL can directly invade the digestive tract mucosa, causing its damage and triggering inflammatory lesions (9, 10). In this case, the patient underwent three colonoscopies, which found an ileocecal ulcer with active bleeding. At the same time, VL caused HLH and hypersplenism, leading to significant thrombocytopenia (with a platelet count as low as 45 × 10^9^/L) and coagulation dysfunction (the maximum D-Dimer level reached 41.87 mg/L, and the minimum fibrinogen level was 1.22 g/L), which ultimately led to recurrent bleeding.

Like intestinal hemorrhage, VL-associated HLH is equally rare and life-threatening. In contrast to EBV, a common trigger for HLH, VL is a rare factor, that predominantly affects immunocompromised individuals. However, patients with VL-associated HLH presented increased risks of bleeding, organ dysfunction, and inflammatory cytokine levels, along with elevated parasite loads, compared to those with VL alone (11). Chronic infection with Leishmania leads to the uncontrolled activation of cytotoxic lymphocytes and macrophages. The persistent activation of these cells leads to an excessive cytokine storm that triggers HLH, which may lead to multiorgan failure and high mortality (12). Qin et al. (13) summarized the cases of 111 patients diagnosed with HLH through 2022, a total of 18.4% of patients died of VL-associated HLH, mainly due to infection, bleeding, or multiple organ failure. Generally, specific management of the underlying trigger of HLH is often essential to eliminate the excessive immune activation that drives its development. In this case, the patient tested negative for EBV on admission (March 18) but already exhibited typical manifestations of HLH. Subsequent viral load testing on March 27 indicated an EBV-DNA of 9.79 × 10^3^ IU/mL, prompting of a 12-day course of acyclovir treatment. During treatment, the recurrent fever remained unresolved and laboratory indicators continued to deteriorate, presenting a contradiction with the seronegative EBV viral load. These findings strongly suggested that despite the presence of EBV infection, VL remains the core factor for HLH. Moreover, in accordance with the HLH-1994/2004 treatment guidelines, we initiated a therapeutic regimen combining VP-16 with dexamethasone to reduce the cytokine storm and excessive immune responses, and further improve the patient’s symptoms (12).

Untreated symptomatic VL is almost always fatal. Thus, all symptomatic patients with VL need treatment with antileishmanial drugs. The primary pharmacological options against Leishmania include amphotericin B, antimonials, paromomycin, and miltefosine (14). For decades, sodium stibogluconate (SSG) has been considered the mainstay of therapy for leishmaniasis. However, owing to its multiple toxicities and increasing resistance, along with the general unavailability of antimonials in clinical hospitals of non-endemic regions, its position has been supplanted by amphotericin B (15). Amphotericin B used for treating VL is divided into two major categories: traditional deoxycholate formulations (AmB-D) and lipid formulations (L-AmB). The latter exhibits significantly fewer adverse events than AmB-D. Accordingly, it is now increasingly recognized as the first-line treatment in regions including the Indian Subcontinent, the Mediterranean area, and the Americas. However, a significant drawback of L-AmB is the economic burden, which remains unaffordable for most leishmaniasis-endemic countries (14, 16). In an open-label Indian study, AmB-D was not inferior to L-AmB, achieving a 98% cure rate in 108 VL patients (17). Although IDSA guidelines recommend L-AmB as the first-line medication, proper usage of AmB-D may be the better option in terms of cost and availability in limited areas and regions. However, AmB-D is associated with myelosuppression, high nephrotoxicity and hypokalemia, and its side effects should be closely monitored during treatment (18, 19).

## 4 Summary

In conclusion, we admitted a patient with VL accompanied by systemic multiorgan failure. The patient was ultimately diagnosed with VL through mNGS and received AmB-D treatment. During the hospitalization, the patient developed two rare complications: VL-associated HLH and intestinal hemorrhage. Throughout the treatment process, we collaborated with experts from the departments of gastroenterology, radiology, hematology, and gastrointestinal surgery via a multidisciplinary approach to ensure the selection of a comprehensive and timely therapeutic approach for the patient. Through the collective efforts of the team, the patient achieved a favorable long-term prognosis.

## Data Availability

The original contributions presented in the study are included in the article/supplementary material, further inquiries can be directed to the corresponding authors.
